# Negative Mood State Enhances the Susceptibility to Unpleasant Events: Neural Correlates from a Music-Primed Emotion Classification Task

**DOI:** 10.1371/journal.pone.0089844

**Published:** 2014-02-28

**Authors:** Jiajin Yuan, Jie Chen, Jiemin Yang, Enxia Ju, Greg J. Norman, Nanxiang Ding

**Affiliations:** 1 Key Laboratory of Cognition and Personality of Ministry of Education, School of Psychology, Southwest University, Chongqing, China; 2 Research Center for Psychological Development and Education, Liaoning Normal University, Dalian, Liaoning, China; 3 Chongqing Three-Gorges Normal School, Wanzhou, Chongqing, China; 4 Department of Psychology, Center for Cognitive and Social Neuroscience, University of Chicago, Chicago, Illinois, United States of America; Vanderbilt University, United States of America

## Abstract

**Background:**

Various affective disorders are linked with enhanced processing of unpleasant stimuli. However, this link is likely a result of the dominant negative mood derived from the disorder, rather than a result of the disorder itself. Additionally, little is currently known about the influence of mood on the susceptibility to emotional events in healthy populations.

**Method:**

Event-Related Potentials (ERP) were recorded for pleasant, neutral and unpleasant pictures while subjects performed an emotional/neutral picture classification task during positive, neutral, or negative mood induced by instrumental Chinese music.

**Results:**

Late Positive Potential (LPP) amplitudes were positively related to the affective arousal of pictures. The emotional responding to unpleasant pictures, indicated by the unpleasant-neutral differences in LPPs, was enhanced during negative compared to neutral and positive moods in the entire LPP time window (600–1000 ms). The magnitude of this enhancement was larger with increasing self-reported negative mood. In contrast, this responding was reduced during positive compared to neutral mood in the 800–1000 ms interval. Additionally, LPP reactions to pleasant stimuli were similar across positive, neutral and negative moods except those in the 800–900 ms interval.

**Implications:**

Negative mood intensifies the humans' susceptibility to unpleasant events in healthy individuals. In contrast, music-induced happy mood is effective in reducing the susceptibility to these events. Practical implications of these findings were discussed.

## Introduction

Various affective disorders, such as depression, anxiety, and PTSD (Post-traumatic stress disorder), are known to be associated with enhanced brain processing of unpleasant stimuli [1]–[5]. In early studies, researchers used a variety of behavioral tasks, such as dot-probe and color-naming tasks, to find that clinically anxious individuals were equipped with a biased attentional sensitivity for threatening stimuli [3], [6], [7]. These results were confirmed by later event-related potential studies displaying enhanced early potential responding to threat-related stimuli in highly anxious individuals [5], [8]. Moreover, in addition to reports of reduced sensitivity to pleasant stimuli [9], [10], many studies have reported that depressed individuals display a sustained processing bias for unpleasant words [11], faces [2], [5] and pictures [1], possibly as a result of dysfunction in suppressing unpleasant information [1]. Furthermore, studies of post-traumatic stress disorders (PTSD) consistently showed that PTSD symptom measures (e.g. arousal and avoidance), were effective in predicting the intensity of brain responding to unpleasant, trauma-related threatening, stimuli [12], [13]. Similar patterns of association were also observed for other disturbances, such as various kinds of phobia and dysphoria disorders [14]–[17].

Nevertheless, evidences from a few studies implied that the enhanced processing of unpleasant stimuli may not be a result of the disorder itself. Instead, this association may result from the predominant negative mood states derived from these disorders [4], [18]. For instance, Mathews et al [3] observed that threatening stimuli interfered with task processing more intensely in Generalized Anxiety Disorder patients compared to control individuals. However, this group difference vanished when patients received a therapy that reduced state anxiety [3]. This likelihood was supported by recent findings that unpleasant stimuli triggered greater attention bias in state, but not in trait, anxiety populations [4], [19]. In fact, using healthy subjects, a few studies have explored how mood influences emotion perception from ambiguous faces [20], [21]. The results showed that subjects perceived more sadness/rejection from these faces, and tended to evaluate these faces as unpleasant, when they were experiencing induced sadness or depression [20]–[22]. In addition, a recent study investigated the influence of mood on the strength of emotional negativity bias using ERP measures [23]. The results showed enhanced processing of negative over positive stimuli in both sad and happy mood states, while the strength of negative bias was increased during sad versus happy moods.

However, these studies used ambiguous facial expressions that were not emotionally salient. Without using emotionally salient materials, this approach is unable to clarify how mood state impacts the brain's sensitivity to emotional events. In addition, there was evidence showing that facial expressions involve more emotional perception or recognition rather than emotion responding [24]. In contrast, emotionally salient pictures that depict stressful or pleasurable real-life scenes [25], have been verified effective to elicit emotion experience and visceral-physiological activations, such as heart-rate and skin conductance changes that validly predict subjective emotion arousal [24], [26]. Based on this feature, emotional pictures are often used to study brain susceptibility to emotional events [19], [27], and this susceptibility was recently considered a predictor for wellbeing and mental health [28]. Though our prior study addressed how mood impacts negative bias using emotional pictures [23], the focus on negative bias which entails negative-positive comparison and the lack of a neutral mood, made it unlikely to conclude how mood state modulates the susceptibility to unpleasant events [12], [29]. Thus, a direct study is necessary, to clarify how mood state impacts the susceptibility to unpleasant stimuli in healthy populations.

Therefore, the present study investigated the impact of mood state on brain's susceptibility to unpleasant events using emotionally salient pictures. We used happy or sad music excerpts for mood induction, as music has been verified effective to induce a stable and intense mood state [30]–[32]. Previous studies also used static scenes, faces or guided imagination to induce emotional context [33]–[35]. Despite effectiveness in constructing emotional context and inducing short-lived emotion, these approaches are not suitable for inducing a sustained and stable mood state. For example, though guided imagination elicits a significant positive mood compared to pre-induction, the mood intensity was reduced at the end of the task [34]. By contrast, music has a great power of inducing a stable mood state: music is able to elicit intense pleasant and unpleasant experiences [31], [36], increased peripheral physiological activities (e.g. heart and respiration rates) as well as broad activations of limbic/paralimbic circuits including ventral striatum, midbrain, orbitofrontal cortex,amygdala and insula [31], [36], even in the absence of explicit tasks [37]. On the other hand, there were a growing number of studies that reported an influence of musical listening on the processing of visual emotional and neutral targets in both healthy, psychopathic and musician populations [38]–[41]. For example, the evaluation of emotional visual stimuli, whether faces or words, was facilitated when paired with emotion-congruent relative to incongruent music pieces in musician, non-musician and Williams syndrome populations [38], [41]–[43]. Based on these evidences, it is apparent that music excerpts are excellent materials to elicit stable mood states and to investigate the impact of mood state on brains' susceptibility to emotional stimuli. Thus, the present study used Chinese classic music, which are familiar to local subjects, as mood-inducing materials to ensure the quality of mood induction [44]. Instead of requiring pleasant/unpleasant distinction, the present study required subjects to classify the picture as emotional or neutral, irrespective of the valence, consequently to avoid a task-relevant effect that obscures the emotion effects in brain potentials [45].

Prior studies showed that the negative bias of anxious individuals was accounted for by the enhanced state instead of trait anxiety [4], [19], and that healthy subjects perceived more sadness from ambiguous faces during induced depression [21]. In addition, it was consistently reported that listening to sad music enhanced the perceived sadness from the faces [42], [43]. Based on these evidences, we hypothesized that the induction of negative mood state in healthy subjects may increase neural responding to unpleasant stimuli in comparison with the neutral mood. On the other hand, because prior studies showed reduced attention bias for negative stimuli in the pleasant context, and less perception of sadness from faces during happy music [42], [46], we predict that the susceptibility to unpleasant stimuli may decrease under positive mood induction.

Specifically, many studies have revealed that Late Positive Potential (LPP), a central- parietal positive slow ERP that reaches largest amplitudes 500–700 ms post-stimulus and lasts for several hundred milleseconds, was more pronounced for emotionally salient than for neutral stimuli [47]–[50]. In addition, LPP amplitudes were accepted as a valid index for the strength of emotion arousal: the LPP amplitudes decreased with the reduction of experienced emotion arousal, and increased with the enhancement of the arousal [47]–[50]. Thus, we firstly predict that unpleasant pictures elicit enhanced LPP amplitudes compared to neutral pictures in the neutral mood condition. More importantly, the emotion effect for unpleasant pictures, indexed by unpleasant –neutral differences in LPP amplitudes, may be enhanced during music-induced negative mood, while this effect may be decreased during music-induced positive mood condition.

Lastly, previous studies on affective priming consistently reported an emotional congruency effect when the task required assessing affective valence of the target stimuli [41], [42], [51], [52] or assessing affective congruency between the prime and the target [52]. Specifically, the emotional valence (i.e. pleasant or unpleasant) of the target stimulus (e.g. emotional words) was evaluated faster when preceded by emotion-congruent primes (e.g. music) than when preceded by emotion-incongruent primes [41], [51], [53]. This emotional congruency effect was also embodied by a pronounced N400 effect in brain potentials 400–500 ms post target onset, and reduced superior temporal gyrus but increased fusiform gyrus activations, during emotion-incongruent than during congruent conditions [41], [42], [51]. However, these affective congruency effects disappeared, “neither observed at the behavioral nor electrophysiological level”, when the task did not require subjects to assess the emotional valence (positive or negative) of target stimuli [53]. Therefore, we predict that there would not be a similar emotional congruency effect during our task, which does not require subjects to assess the valence of the pictures.

## Materials and Methods

### 2.1. Subjects

As paid volunteers, 16 native Chinese students (8 women, 8 men) with a mean age of 22.06 years (range: 20–24years) participated in the study. All subjects were healthy, right-handed, with normal or corrected to normal vision and audition, and reported no history of affective disorder. Each subject signed an informed consent form for the experiment, and filled in the Zung Self-rating Depression/Anxiety Scales [54], [55]. The mean scores for SDS and SAS measures were 37.3 (S.D. = 2.5) and 36.5 (S.D. = 2.1), respectively, and all of them were within the normal range of the healthy population. The experimental procedure was in accordance with the ethical principles of the 1964 Declaration of Helsinki (World Medical Organization, 1996). The study was approved by the Review Board for Human Participant Research, School of Psychology of Southwest University (China). Each subject signed an informed consent form prior to the experiment.

### 2.2. Stimuli

The stimulus materials consisted of 480 prime–target pairs. Mood-inducing music excerpts were used as the primes. Pictures from the native Chinese Affective Picture System were used as the targets [56], [57]. The pictures covered a variety of contents. These stimuli were primarily composed of the three categories: animals (e.g. snakes, eagles, and pandas), natural scenes (e.g. fire disaster, clouds, and landscapes) and human activity (e.g. violence, sports, and cheers). We used a block design, and each block presented a single category of mood-inducing excerpts. The purpose of this method was to improve the quality of mood induction and maintain the homogeneity of the mood in each block [28], [29]. According to the mood, the experiment was divided into 3 blocks: positive, negative and neutral mood blocks. The targets in each block were composed of 40 pleasant (25%), 40 unpleasant (25%) and 80 neutral (50%) pictures. Because we used an emotional/neutral classification task instead of a valence evaluation task, the present study used neutral pictures twice as many as pleasant or unpleasant pictures in each block, consequently to equate the onset frequency of emotional and neutral stimuli.

Mood-inducing materials consisted of 40 sad musical excerpts, 40 happy musical excerpts and 40 neutral broadcast excerpts. Happy music materials were made of instrumentally played Chinese folk music like “Crave for the Red Army” which was characterized by cheerful rhythm and light-hearted, elated melodies. Also, sad music excerpts were made of the instrumental Chinese folk music like “Two Springs Reflect the Moon” that was played at a slow pace and was characterized by sad, depressed melodies. Because both happy and sad music excerpts were played by instruments, free of verbal words, we used Mongolian broadcast sentences whose meanings were unknown to the subjects as the baseline neutral excerpts, consequently to induce a neutral mood free of semantic contamination. All excerpts were matched for the sound intensity, and were digitized with a 44.1-kHz sampling rate and 16-bit resolution using the CoolEdit Pro software. Another sample of non-musician college students (n = 50, 25 men; 18–24years), who did not participate in the ERP experiment, were recruited to rate the valence (from 1 = ‘extremely unpleasant’ to 9 = ‘extremely pleasant’) and arousal (1 = ‘very calm, relaxed’ to 9 = ‘very frenzied, excited’) of these excerpts using a 9-point scale. The results showed significantly higher valence ratings for happy excerpts (M = 7.11; *SD* = 0.73) vs. neutral (M = 4.61; *SD* = 0.81; p<.001) excerpts whose valence scores, in turn, were significantly higher than those of sad excerpts (M = 3.07;*SD* = 0.76; p<.001; the main effect: F(2,119) = 350.5, p<.001). In addition, happy (M = 5.9;*SD* = 1.48) and sad (M = 5.75;*SD* = 1.05) excerpts, which obtained similar arousal ratings [F(1,79) = 2.86, p = .10], were both more arousing in comparison with neutral excerpts(M = 3.95;*SD* = 1.58; the main effect: F(2,119) = 411.56, p<.001). Prime materials were controlled for the length of presentation. Happy music excerpts lasted for 11.52 s, sad excerpts lasted for 11.94 s and neutral excerpts lasted for 11.33 s (F(2,119) = 1.64; P = .20). Lastly, two judges (1 female) naive to our experimental purposes were invited to name the mood induced by each music excerpt by selecting an appropriate description from the following 7 choices: sad, disgust, fear (3 negative choices); happy, pride and serene (3 positive choices); and neutral. The results showed that both judges selected “sad” for each of 40 sad musical excerpts, and “happy” for each of 40 happy excerpts. Thus, the happy musical, sad musical and neutral broadcast excerpts used by the present study were valid to induce happy, sad and neutral mood states.

The pictures used for this study were selected in such a way that the pleasant (*M* = 7.31, *SD* = 0.29), neutral (*M* = 5.27, *SD* = 0.48) and unpleasant picture sets (*M* = 1.91, *SD* = 0.31) differed significantly from one another in valence (all p<.001; the main effect: F(2,239) = 2809.63; P<.001). In addition, pleasant (*M* = 5.97, *SD* = 0.62) and unpleasant (*M* = 6.16, *SD* = 0.86) pictures, which were similar in arousal (F(1,19) = 1.92; P = .17), were both more arousing than neutral pictures (*M* = 3.8, *SD* = 0.66; both p<.001; the main effect: F(2,239) = 307.88; p<.001). Moreover, the valence and arousal of each picture category were kept highly similar across the three blocks, in order to attribute the results from dependent variables solely to mood induction. Unpleasant pictures were 1.91+0.32(or 6.18+0.92) for the sad mood block, 1.91+0.32(or 6.15+0.90) for the happy mood block, and 1.91+0.32(or 6.16+0.83) for the neutral mood block in valence (or, arousal) (all p>0.80). Neutral pictures were 5.27+0.46 (3.82+0.73) for the sad mood block, 5.27+0.51(3.78+0.57) for the happy mood block, and 5.27+0.47(3.81+0.70) for the neutral mood block in valence (or, arousal) (all p>0.80). Pleasant pictures were 7.30+0.27 (5.99+0.63) for the sad mood block, 7.32+0.28(6.01+0.63) for the happy mood block, and 7.32+0.32 (5.91+0.61) for the neutral mood block in valence (or arousal) (all p>0.60).

### 2.3. Procedures

**Figure 1:**
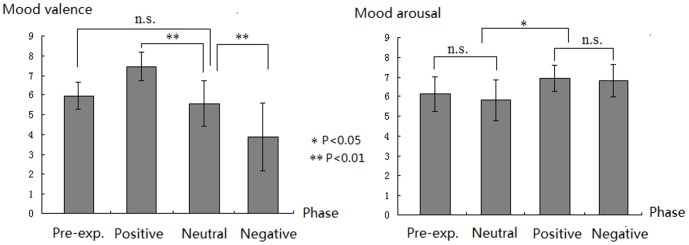
The results of the mood valence and arousal ratings for the pre-experiment phase, and for neutral, positive and negative mood induction phases. Error bars represent the standard deviation (SD).

Subjects were seated in a quiet room at approximately 150 cm from a computer screen with the horizontal and vertical visual angles below 5°. Each trial was initiated by a 300 ms presentation of a small white cross on the black computer screen. Then, a musical/broadcast excerpt was presented via an earphone, whose offset was then followed by a variable blank screen for 300–600 ms. Subsequently, a target emotional image was presented for 1000 ms. Half of the subjects were instructed to press the “F” key on the key board as quickly as possible if an emotional (pleasant or unpleasant) picture was presented, and to press the “J” key if a non-emotional, neutral picture was presented. The response hands were reversed for the remaining subjects. The picture presentation was terminated by a key pressing, or was terminated when it elapsed for 1000 ms. Therefore, each subject was informed that their responses must be made under 1000 ms. Each response was followed by 1500 ms of a blank screen. The sequence of pleasant, unpleasant and neutral pictures was randomized for each subject. To avoid the fatigue effect that may contaminate the results, each block was divided into 8 sections, and the end of each section allowed subjects about 30 s to rest. The order of three blocks was balanced across subjects. Before the experiment and at the end of each block, subjects were asked to rate the valence (1 = ‘extremely unhappy’ to 9 = ‘extremely happy’) and arousal (1 = ‘not arousing at all’ to 9 = ‘extremely arousing’) of their mood using a nine-point scale (for the results, see Fig. 1).

### 2.4. ERP recording and analysis

Brain electrical activity was recorded at 64 scalp sites using tin electrodes mounted in an elastic cap (Brain Product, Munchen, Germany), with references on the left and right mastoids and a ground electrode on the medial frontal aspect (average mastoid reference, [58].The impedance of all electrodes was less than 5 kΩ. Vertical electrooculograms (EOGs) were recorded supra-orbitally and infra-orbitally from the left eye. The horizontal EOG was recorded as the left versus right orbital rim. The EEG and EOG were amplified using a 0.05–100 Hz bandpass and continuously digitized at 500 Hz/channel for offline analysis. The data were offline filtered using a bandpass filter (0.1–24 Hz). The averaging of ERPs were computed off-line using the Vision Analyzer software (Brain Products, Munich, Germany). EOG artifacts (blinks and eye movements) were corrected using the eye movement correction algorithm recommended by Gratton and colleagues [59]. Artifact-free EEG segments to trials with correct responses were averaged separately for each experimental condition. Trials with EOG artifacts (mean EOG voltage exceeding ±80V),amplifier clipping artifacts, or peak-to-peak deflection exceeding ±80V were excluded from averaging. The rejected trials were rare, and there were enough trials obtained for ERP averaging in each subject and each condition. The averaged number of trials was 62.81 for neutral, 35.75 for pleasant, and 35.62 for unpleasant pictures in the positive mood block. In the negative mood block, the averaged number of trials was 65.94 for neutral, 32.69 for pleasant, and 35.06 for unpleasant pictures. In the neutral mood block, there were 63.31 trials, 35.63 trials, and 36.75 trials on average for neutral, pleasant and unpleasant picture sets, respectively.

**Figure 2:**
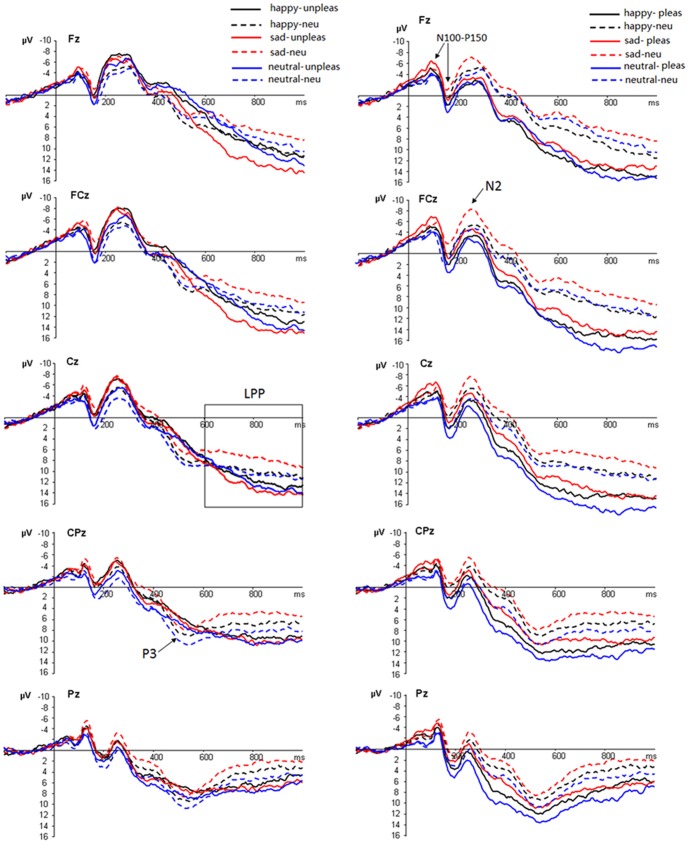
Left: Averaged ERPs at Fz, FCz, Cz, CPz and Pz for unpleasant (solid lines) and neutral (dashed lines) pictures during positive (happy, black lines), negative (sad, red lines) and neutral (blue lines) mood states. Right: Averaged ERPs for pleasant (solid lines) and neutral (dashed lines) pictures during positive (black lines), negative (red lines) and neutral (blue lines) mood states.

**Figure 3:**
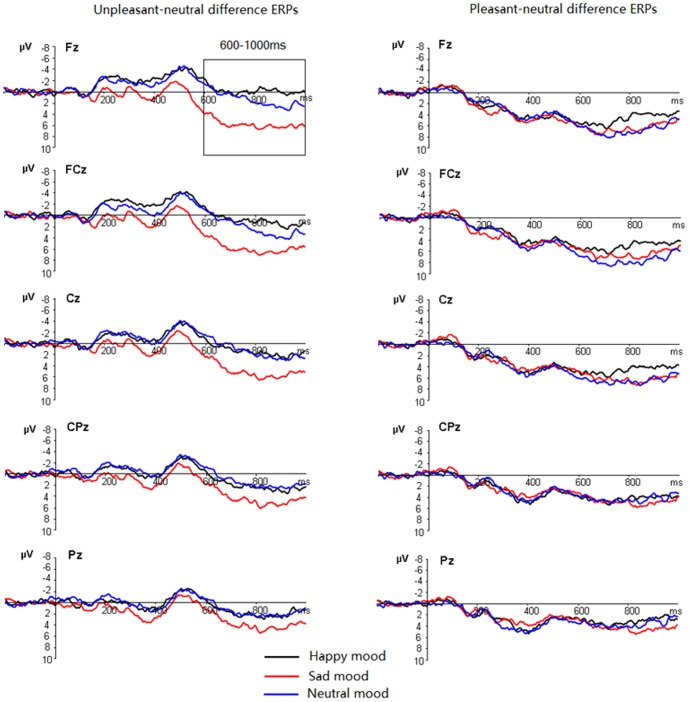
Left: the unpleasant picture-neutral picture difference waves in the positive (black lines), negative (red lines) and neutral (blue lines) mood states at Fz, FCz, Cz, CPz and Pz. Right: the pleasant picture-neutral picture difference waves in the positive, negative and neutral mood states at these sites.

ERP waveforms were time-locked to the onset of picture stimuli and the average epoch was 1200 ms, including a 200 ms pre-stimulus baseline. The following 25 electrode sites were selected for statistical analysis: F_3_, F_1_, F_z_, F_2_, F_4_ (5 front sites); FC_3_, FC_1_, FC_z_, FC_2_, FC_4_ (5 frontocentral sites); C_3_, C_1_, C_Z_, C_2_, C_4_ (5 central sites); CP_3_, CP_1_, CP_Z_, CP_2_, CP_4_ (5 centroparietal sites); P_3_, P_1_, P_Z_, P_2_, P_4_ (5 parietal sites). As shown by the averaged ERPs and their topographical distributions (Fig. 2), there were prominent N100 (70–130 ms), P150 (130–200 ms) and N2 (220–300 ms) components that were largest across central- to-frontal scalp regions. In addition, there was a parietal P3 component in the 450–550 ms interval, and a late positive potential (LPP) that spans the 600–1000 ms interval (Fig. 2 and 3). Previous studies considered N100 and P150 components as indexes of early sensory- perceptual processing that attends to salient stimulus features [18], [60], [61]. Thus, we analyzed the N100 and P150 components as a N100-P150 complex, using a peak-to-peak method to increase the signal to noise ratio (for an analysis, see [62]). Specifically, we quantified N100-P150 amplitudes as the peak-to-peak voltage differences between the most positive and the most negative peaks in the 70–200 ms interval [62], [63]. In addition, we measured peak latencies and peak amplitudes of the N2 in the 220–300 ms interval. Peak latencies are defined as the duration from stimulus onset to the time point when the component reached peak amplitudes. Peak amplitudes are quantified as the peak amplitude values against baseline. Because the P3 component showed no visible peaks for emotional pictures and the LPP was slow positive waves, we measured the mean amplitudes of the P3 and LPP in the 450–550 ms and 600–1000 ms intervals, respectively. A four-way repeated measures analysis of variance (ANOVA) was conducted for each of these components. ANOVA factors were mood state (3 levels: positive, negative & neutral), picture category (3 levels: pleasant, unpleasant and neutral), frontality (5 levels: frontal, frontocentral, central, centroparietal & parietal) and laterality (5 levels: left, midline-left, midline, midline-right, right). In order to explore the timing features of LPPs, we added timing (4 levels: 600–700 ms, 700–800 ms, 800–900 ms; 900–1000 ms) as another factor to the ANOVA model during LPP analysis, as recommended by [47], [50], [64]. The results of latency analyses for these components were not reported, because there were no significant main or interaction effects produced by mood, picture valence, or electrode sites factors.

In order to assess the emotional congruency effect, we averaged the P3 (or LPP) amplitudes across happy music-positive picture and sad music-negative picture conditions to compute the ERPs for the congruent condition. Similarly, we averaged the amplitudes across happy music-negative pictures and sad music-positive pictures to compute ERPs for the incongruent condition, an approach used by Kamiyama et al., [52] and Goerlich, et al., [51]. The emotional congruency effect in the P3 and LPP amplitudes was then tested by a paired-sample t test. The degrees of freedom of the *F*-ratio were corrected according to the Greenhouse-Geisser method, and Fisher's Least Significant Difference (LSD) method was used for post hoc pairwise comparisons if significant main or interaction effects were detected.

## Results

### 3.1. RT & Accuracy

Unpleasant pictures (*M* = 96.3%) obtained more accurate classification than pleasant pictures (93.3%, *p*<.01) which, in turn, was classified more accurately than neutral pictures (87.9%, *p*<.05), irrespective of mood state (*F*(2,30) = 11.25, *p*<.005).No other effects were detected in accuracy data. The analysis of reaction times (RT) with correct responses demonstrated faster RTs for pleasant pictures (*M* = 630 ms) than for unpleasant (652 ms, F(1, 15) = 10.03; *p*<.01) and neutral [671 ms; F(1, 15) = 9.28, *p*<.01] pictures, irrespective of mood [*F*(2, 30) = 5.56, *p*<.03]. The RT differences between unpleasant and neutral pictures were not statistically significant (F(1, 15) = 1.60, p = .23).No other effects were detected in RT analysis.

### 3.2 Mood Rating

Mood valence and arousal ratings were analyzed by a repeated measures ANOVA with four levels of mood induction phases: pre-experiment (no induction), neutral, negative and positive mood inductions. Valence ratings differed significantly depending on the induction phase [F(3, 45) = 35.08, p<.001; η2_p_ = 0.70]. The valence rating score was higher in the positive mood (M±SD: 7.44±0.73) compared to the neutral mood [ t(15) = 6.54, p<.001] and the pre-experimental [t(15) = 7.35,p<.001] phases. In addition, the valence rating scores were higher in the neutral mood [t(15) = 4.06, p = .001] and the pre- experiment [t(15) = 4.77,p<.001] phases compared to the negative mood phase (3.88±1.71). The mood valence ratings were not significantly different during neutral mood (5.56±1.05) and the pre-experiment (5.94±0.89) phases [t(15) = 1.38, p = .19].

The mood arousal ratings were significantly different across the four mood induction phases [F(3,45) = 10.43, p<.001, η2_p_ = 0.41]. Positive (6.94±0.68) and negative (6.81±0.83) mood blocks elicited higher mood arousal than the neutral block[t(15) = 7.27, p<.001; t(15) = 3.16, p = .006]. However, the arousal rating scores were similar for the positive and negative mood phases [t(15) = 0.62; ns]. Also, the mood arousal was not significantly different between pre-experimental (6.13±0.89) and neutral (5.81±1.05) mood phases [t(15) = 1.43, p = .17]. Thus, the results of mood valence and arousal ratings consistently showed that the mood induction procedure used in the present study was effective in inducing corresponding positive, negative and neutral mood states (see Fig. 1).

### *3.3.* ERP: N100-P150 complex (70–200 ms)

Pleasant pictures (9.38 µV) elicited enhanced amplitudes compared to unpleasant (6.78 µV; F(1,15) = 46.46;p<.001) and neutral pictures (6.68 µV; F(1,15) = 40.45; p<.001), and this effect was most pronounced at midline central-frontal sites (e.g. FCz, Cz). In addition, positive (7.05 µV) mood, but not negative mood (7.62 µV), was associated with smaller amplitudes in comparison with the neutral mood (8.17 µV, p<.001), irrespective of picture valence (Table 1). No other significant effects were detected.

**Table 1:** The results of ANOVA for the amplitudes of N100-P150, N2, P3 and LPP components.

	mood		picture valence		frontal		laterality		frontal*mood		frontal*picture valence		mood*picture valence		frontal*mood *picture valence	
	*F*	*P*	*F*	*P*	*F*	*P*	*F*	*P*	*F*	*P*	*F*	*P*	*F*	*P*	*F*	P
N100-P150	5.53	0.016	33.85	0.0001	0.488	0.55	1.67	0.21	1.02	0.39	2.79	0.058	1.13	0.35	1.96	0.11
N2	8.06	0.002	26.3	0.0001	24.1	0.0001	11.15	0.0001	2.04	0.13	14.43	0.0001	1.72	0.19	1.29	0.28
P3(450–550 ms)	1.11	0.34	34.48	0.0001	24.04	0.0001	1.82	0.18	3.44	0.026	15.99	0.0001	1.51	0.23	1.42	0.22
LPP(600–1000)	4.32	0.025	41.88	0.0001	17.65	0.0001	1.39	0.26	1.31	0.29	13.2	0.0001	4.14	0.013	3.98	0.004

### 3.4. ERP: N2 (220–300 ms)

The amplitudes were more pronounced during positive (−4.78 µV, p<.02) and negative (−5.47 µV, p<.005) compared to neutral (−3.63 µV) mood blocks. Frontal and central sites recorded larger amplitudes compared to parietal sites (see fig. 2), and the scalp midline sites (−5.74 µV) showed larger amplitudes than the left (−4.19 µV) and the right (−3.43 µV) regions. In addition, unpleasant pictures (−5.76 µV) elicited enhanced amplitudes than neutral pictures (−4.91 µV, p<.001) which, in turn, elicited enhanced amplitudes than pleasant pictures (−3.22 µV, p<.001). This effect was more pronounced in frontal [F(2,30) = 37.34, p<.001] and central sites [F(2,30) = 21.02, p<.001] in comparison with parietal sites [F(2,30) = 9.52, p = .001].

### 3.5. ERP: P3 (450–550 ms)

Pleasant pictures (9.33 µV) elicited enhanced amplitudes than neutral pictures (6.02 µV, p<.001) which, in turn, elicited enhanced P3 amplitudes than unpleasant pictures (3.74 µV, p<.001). The P3 amplitudes were larger at parietal (8.76 µV) than at central (6.50 µV, p<.005) and frontal (3.25 µV, p<.001) sites. Whereas the P3 was more pronounced for pleasant than for neutral pictures across all frontality levels (see fig. 2), the P3 amplitudes were larger for neutral versus unpleasant pictures at central and frontal [F(1, 15) = 12.30, p<.005], but not in parietal [F(1, 15) = 2.27, p = .15] sites. In addition, while the P3 was more pronounced for pleasant than for neutral pictures across all laterality levels, P3 amplitudes were larger for neutral versus unpleasant pictures at left [F(1, 15) = 11.79, p<.005] and midline [F(1, 15) = 5.94, p<.03] but not in the right [F(1, 15) = 2.71, p = .12] regions. Furthermore, Positive (8.12 µV) and negative (7.72 µV) moods were linked with smaller P3 amplitudes relative to neutral mood (9.44 µV, p_s_<.05) in centraparietal and parietal sites [F(2, 30) = 4.24, p<.03] but not in central and frontal sites [F(2, 30) = 0.18,ns].

The analysis of the affective congruency effect showed no significant amplitude differences between congruent (6.77 µV) and incongruent (5.97 µV) conditions in 450–550 ms time window (t(15) = 2.11, p>0.05).

### 3.6. ERP: Late Positive Potentials (LPP)

The LPP amplitudes were larger at central (10.91 µV) and centrofrontal (11.18 µV) sites compared to parietal (5.29 µV) sites (table 1). There was a significant four-way interaction amongst frontality, timing, mood and picture valence (F(48,720) = 2.08, p = .012; η2_p_ = 0.12). In order to disentangle the four-way interaction, we tested the significance of the frontality, mood and picture valence interaction in each of the four time windows.

#### 600–700 ms

The three-way interaction was significant (table 1). This effect resulted from the significant mood and picture valence interaction at frontal (F(4,60) = 7.06, p<0.001), frontocentral (F(4,60) = 5.17, p = .005) but not in central, centroparietal and parietal regions (F(4,60) = 0.71–2.64, p = .072–.54). Therefore, the mood by picture valence interaction was then decomposed across frontal and frontocentral regions. The results showed larger amplitudes for negative compared to neutral pictures in the negative mood (t(15) = 3.92, p = .001), but not during positive (t(15) = −1.06, p = .31) and neutral mood (t(15) = −0.49, p = .63) states. The strength of the unpleasant effect, indicated by the negative-neutral amplitude differences, was more pronounced during negative (3.81 µV) than during positive (−0.95 µV) and neutral moods (−0.40 µV; F(2,30) = 10.96, p = .001, η2_p_ = 0.42). On the other hand, positive pictures elicited larger amplitudes than neutral pictures during positive, neutral and negative mood states (t(15) = 5.92–9.07; all p<.001). The strength of the pleasant effect was not significantly different across the three mood conditions (F(2,30) = 1.59, p = .22).

#### 700–800 ms

The three-way interaction was significant (Table 1). This effect resulted from the significant mood and picture valence interactions at all regions (F(4,60) = 2.87–8.42, p = .00–.045) except the parietal region (F(4,60) = 0.71, ns). Breaking down the mood by picture interaction across centroparietal and more anterior regions showed significantly larger amplitudes for negative compared to neutral pictures in the negative mood (t(15) = 4.13, p = .001), but not in the positive (t(15) = 1.31, p = .21) and neutral mood (t(15) = 1.38, p = .18) states. The strength of the unpleasant effect, indicated by negative-neutral amplitude differences, was more pronounced during negative (4.47 µV) than during positive (0.76 µV) and neutral mood (1.19 µV; F(2,30) = 7.28, p = .004, η2_p_ = 0.33). On the other hand, positive pictures elicited larger amplitudes than neutral pictures during positive, neutral and negative mood states (t(15) = 6.03–9.59; all p<.001). The strength of the pleasant effect was not significantly different across the three mood conditions (F(2,30) = 1.12, p = .33).

#### 800–900 ms

**Figure 4:**
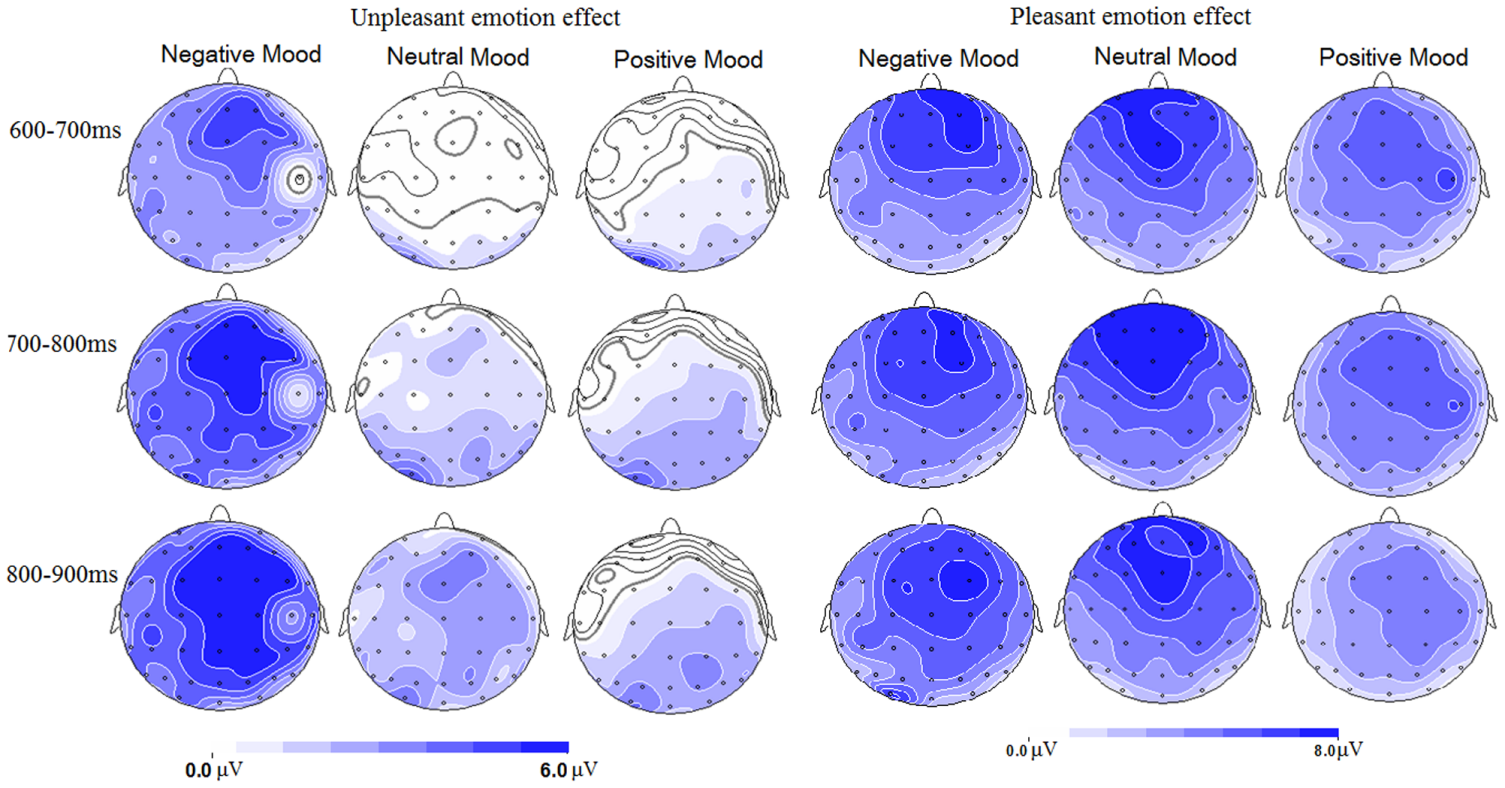
Left: topographical maps of voltage amplitudes for unpleasant picture-neutral picture difference ERPs in the negative, neutral and positive mood blocks in the 600–700 ms, 700–800 ms and 800–900 ms intervals. Right: topographical maps of pleasant picture-neutral picture difference ERPs in the negative, neutral and positive mood blocks in these intervals.

**Figure 5:**
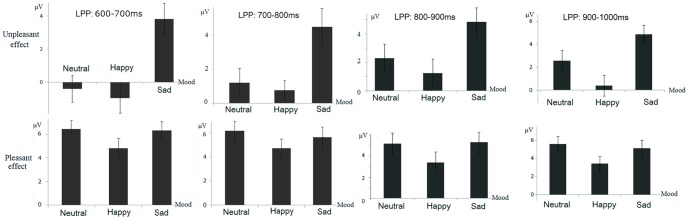
Schematic illustration of the emotion effect for unpleasant (unpleasant-neutral difference amplitudes, Top panel) and pleasant (pleasant-neutral difference amplitudes, Bottom panel) pictures during neutral, positive and negative mood states in every 100 ms of the 600–1000 ms time window. Error bars represent ±standard errors.

The three-way interaction was significant (table 1). This effect resulted from the significant mood and valence interaction at frontal (F(4, 60) = 8.19, p<.001) and frontocentral (F(4, 60) = 4.84, p = .005) regions but not in central and posterior regions (F(4, 60) = 1.32–2.71; all p>.05). Breaking down the interactions in frontocentral and frontal regions showed more positive amplitudes for unpleasant versus neutral pictures during negative (t(15) = 4.94,p = .000] and neutral moods (t(15) = 2.45, p = .027), but not in positive mood (t(15) = −0.14,p = .90). The unpleasant effect, indicated by the amplitude differences between unpleasant and neutral pictures, was more pronounced during negative (5.33 µV) compared to neutral [2.42 µV; F(1, 15) = 7.36, p<.02] mood blocks. The neutral block, in turn, elicited larger size of unpleasant effect than the positive mood block [−0.082 µV; [F(1, 15) = 5.91, p<.03; see Fig. 4 and 5]. In addition, pleasant pictures elicited more positive deflections than neutral pictures during negative, neutral, and positive mood blocks (t(15) = 5.19–7.55/, p_s_<.001]. The strength of the pleasant effect, different from those in the above windows, was significantly compromised during positive (3.56 µV) compared to negative (6.07 µV) and neutral (6.58 µV) mood blocks [F(2, 30) = 4.18, p<.03].

#### 900–1000 ms

The three-way interaction was significant (Table 1). This effect resulted from the significant mood and valence interaction at frontal (F(4, 60) = 6.87, p<.001), frontocentral (F(4, 60) = 2.97, p = .044) regions but not in central and posterior regions (F(4, 60) = 0.95–1.44; p = 0.25–0.42) scalp regions.

Decomposing the interaction effects at frontal and frontocentral regions showed more positive amplitudes for unpleasant versus neutral pictures during negative [t(15) = 6.02, p<.001] and neutral [t(15) = 2.92, p = .011] mood blocks, but not in the positive mood [t(15) = 0.45, p = 0.66]. The unpleasant effect, indicated by the amplitude differences between unpleasant and neutral pictures, was more pronounced during negative (4.85 µV) compared to neutral [2.57 µV; F(1, 15) = 7.74, p = .014] mood blocks. The neutral block, in turn, elicited larger size of the unpleasant effect than the positive mood block [0.40 µV; [F(1, 15) = 4.84, p = .044; see Fig. 4 and 5]. In addition, pleasant pictures elicited more positive ERP deflections than neutral pictures during negative, neutral, and positive moods [t(15) = 4.42–6.21/, p_s_<.001]. The strength of the pleasant effect was not significantly different during positive (3.45 µV), negative (5.17 µV), and neutral (5.60 µV) moods [F(2, 30) = 2.05, p = .15].

Lastly, there was no significant affective congruency effect in LPP amplitudes, as the congruent condition (4.25 µV) elicited similar LPP amplitudes compared to the incongruent (3.72 µV) condition (t(15) = 1.55, p = 0.14).

### 3.7. The timing of the mood effect in LPP responding to negative pictures

The above analyses showed that 1), negative mood intensified LPP reactivity to unpleasant stimuli in every 100 ms interval of the 600–1000 ms time window; 2), positive mood decreased this reactivity in the 800–1000 ms interval but not in the 600–800 ms. To test whether positive and negative moods have different timing features in their influence on negative picture processing, we conducted 2 separate ANOVAs, with mood (2:negative, neutral) * picture (2: unpleasant, neutral) * timing (4 windows) as factors in Model 1; mood (2: positive, neutral) * picture valence (2: negative, neutral)* timing (4 windows) as factors in Model 2. The results showed a significant three-way interaction by Model 2 [F(3,45) = 8.33, p = 0.001; η2_p_ = 0.36] but not by Model 1[F(3, 45) = 0.60, ns]. Thus, the LPP reactivity to unpleasant stimuli was enhanced similarly by negative mood, regardless of timing; while the reduction of LPP reaction to negative stimuli during positive mood was observed just in the 800–1000 ms time windows.

### 3.8. Correlation analysis

**Figure 6:**
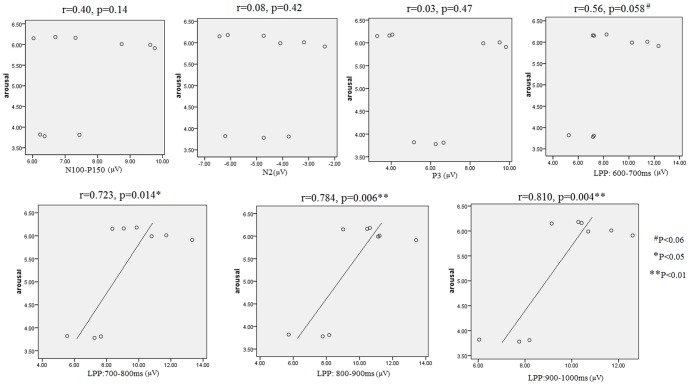
The Scatterplot for the correlation between the pictures' arousal and the amplitudes of each ERP component in the present study. LPP, instead of other components, served as a predictor for the pictures' arousal level.

Firstly, to test whether LPP amplitude was a valid index of subjective emotion arousal, we ran bivariate correlation analyses for the arousal value of each picture set and the LPP amplitudes in the four time intervals. The results showed that LPP amplitudes increased significantly with the arousal of pictures in the 700–800 ms (r = 0.723; p<.02), 800–900 ms (r = 0.784; p<.01), 900–1000 ms (r = 0.810; p<.005); and marginally in the 600–700 ms (r = 0.56; p<.06). Because response times were distributed in the 600–700 ms interval, the LPP of this interval involves response execution that might obscure the interpretation of the correlation in subsequent time intervals. To exclude this confound, we conducted a partial correlation analysis for arousal values and the LPP amplitudes in the 700–800 ms, 800–900 ms, and 900–1000 ms intervals respectively, with the LPP amplitudes of the 600–700 ms as a control variable. The results continued to show larger LPP amplitudes with increasing arousal in the 700–800 ms (r = 0.83, p<.01); 800–900 ms (r = 0.758, p<.02) and 900–1000 ms (r = 0.845, p<.005) intervals (see Fig. 6). To test whether this predicting role was specific to LPP, we further conducted correlation analysis between the picture arousal and the amplitudes of the earlier components. Nevertheless, these analyses failed to generate a significant correlation between the pictures' arousal and the amplitudes of N100-P150 (r = 0.40, p = .14), N2 (r = 0.08, p = .42) and P3 (r = 0.03, p = .47) components (see Fig. 6). Therefore, consistent with many studies [50], [64], the amplitude of LPPs, instead of other components, was a valid predictor for emotion arousal in the present study.

**Figure 7:**
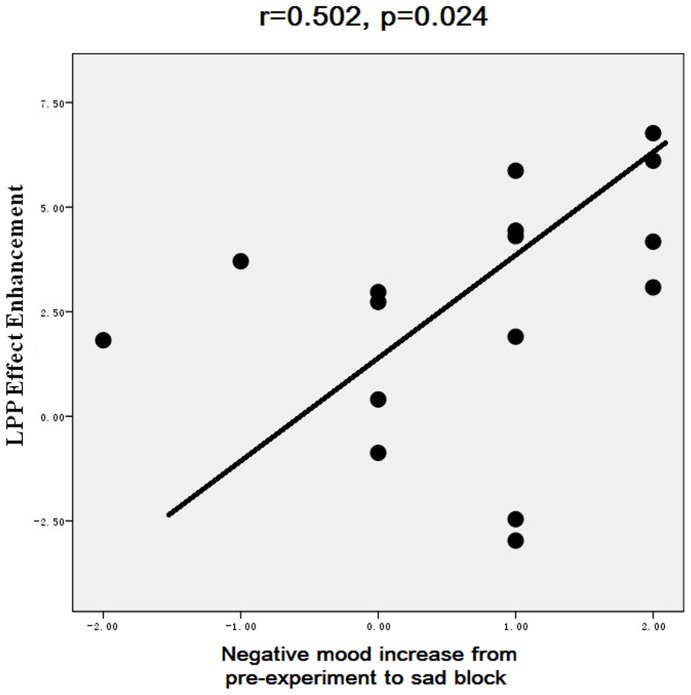
The scatterplot for the correlation between the enhancement of the LPP emotion effect for unpleasant pictures and the arousal of negative mood during sad music. The former was computed by subtracting the unpleasant effect (unpleasant-neutral pictures in the 600–1000 ms) in the neutral block from that in the sad block. The latter was computed by subtracting pre-experiment mood arousal from the mood arousal in the sad block.

Secondly, negative mood state was linked with enhanced LPP responding to unpleasant pictures in the entire 600–1000 ms interval. To verify this observation, we computed the correlation between the enhancement of the LPP effect and the arousal of negative mood during negative mood induction. The enhancement of the LPP effect was computed by subtracting the unpleasant effect (unpleasant- neutral pictures in the 600–1000 ms) in the neutral block from that in the negative mood block. The negative mood arousal was computed by subtracting pre-experiment mood arousal from that in the negative block. The results showed a significant positive correlation(r = 0.502, p = .024, Fig. 7), suggesting that negative mood state enhanced LPP effects for unpleasant pictures to a greater extent with increasing negative mood arousal.

##  Discussion

Using ERP measures and a music-primed picture classification task, the present study observed that the music materials used in this study were effective in inducing negative and positive moods, indicated by the results of music valence/arousal ratings and mood ratings (see 2.2.section and Fig. 1).The ERP data showed significant mood state by picture valence interaction effects on the LPP amplitudes, and LPP amplitudes validly predicted the arousal values of picture stimuli. These interactions were mainly manifested by the increased emotion effect for unpleasant stimuli during negative mood throughout the 600–1000 ms interval, and by the reduced emotion effect for unpleasant stimuli during positive mood in the 800–1000 ms interval.

Our behavioral data showed faster behavioral responses and a higher error rate for pleasant vs. unpleasant pictures, probably as a result of enhanced approach motivation and heightened response tendencies under pleasant stimulation [65], [66]. However, we did not observe a significant mood influence on the processing of affective pictures in behavioral measures. Previous studies in our and other labs consistently showed that response times and accuracy were not as sensitive as electrophysiological measures in revealing brain sensitivity to emotional stimuli [35], [66], [67] and how this sensitivity was influenced by mood context [23]. Thus, the lack of a mood impact on picture processing in behavioral indexes may be explained by that behavioral indexes were not sensitive enough to detect this interaction. This interaction may be observed in electrophysiological levels.

We also observed larger amplitudes in N100-P150 complex for pleasant compared to unpleasant and neutral pictures. Previous studies indicate that humans are equipped with a normative positive mood and a positive expectation for unknown future events that has been suggested to facilitate the processing of a pleasant stimulus [68]–[70]. Therefore, pleasant stimuli, which match this predisposed positive expectation, most likely obtained enhanced perceptual processing that consequently led to larger P150 amplitudes for pleasant versus unpleasant pictures both in the present and our prior studies [23].

In addition, consistent with our prior finding [71], the present study observed larger N2 amplitudes for unpleasant than for pleasant and neutral pictures. This was probably due to the role of the centrally-peaking N2 in indexing vigilance to threatening events [19], [35]. Because neutral and pleasant pictures conveyed no threats, the attention vigilance to these stimuli was probably less intense than that to unpleasant stimuli, thus leading to decreased N2 amplitudes for neutral and pleasant stimuli. Moreover, we observed a parietal P3 component in the 450–550 ms interval that was larger for pleasant than for neutral and unpleasant pictures. Parietal P3 has been accepted as indexing response decisional processes before overt motor action in choice-reaction time tasks [72]–[74]. As such, our observation of larger P3 amplitudes for pleasant compared to neutral/unpleasant pictures should reflect enhanced resources devoted for response decision and motor readiness during pleasant stimulation. This argument was reinforced by our finding of faster motor responses for pleasant vs. other stimuli; and was supported by prior reports that evaluation of positive stimuli is associated with intensified approach motivation and enhanced response tendencies [65], [66]. In addition, we observed smaller P3 amplitudes in negative compared to neutral pictures. Prior studies using valence evaluation tasks consistently reported larger P3 amplitudes for negative compared to neutral pictures [75], [76]. However, several studies showed that negative pictures elicited smaller p3 amplitudes compared to positive and neutral pictures when the task was irrelevant to assessing picture valence [66], [77]. In contrast to the positive stimulus which activates approach motivation, negative stimulus is associated with defensive, avoidant motivation [78]. Thus, one likely explanation is that because positive and negative pictures shared the same response button, the approach motivation initiated by positive pictures should be inhibited and was replaced by avoidant motivation during negative trials. This inhibition may have resulted in smaller p3 amplitudes, consistent with a couple of studies using a similar approach [66], [77]. However, all these components (i.e. N100-P150, N2, and P3) failed to show a significant mood by picture valence interaction, possibly because these components index cognitive steps that mediate performance of picture classification, less relevant to late phases of emotion responding and experience as embodied by late positive potentials (LPP, [47], [50], [79].

The present study observed late positive potentials that span the 600–1000 ms interval, where we observed a significant mood and picture valence interaction in each 100 ms time interval. Late positive potentials, which are sustained slow positive waves that begin after 500 ms and are largest at central sites during affective decision tasks [79], were consistently reported to be larger for emotional versus neutral stimuli [47], [50]. In addition, the LPP amplitudes have been established to reflect the intensity of subjective emotional experience, as many studies observed that LPP amplitude predicted the arousal of self-reported emotion evoked by salient stimuli [50], [64]. Consistent with these findings, the present study observed pronounced LPP activity that began from about 600 ms post picture onset, with their amplitudes largest over central scalp region and their amplitudes more pronounced for emotionally intense than for neutral pictures. Because the frequency of emotional and neutral pictures was equated and response hands counterbalanced, our observation of larger amplitudes for emotional versus neutral stimuli was unlikely a result of handedness or stimulus frequency. In addition, LPP activity occurred in windows (600–1000 ms) most of which went after task completion and response execution (see behavioral data). Therefore, the larger LPPs for emotional vs. neutral pictures in these windows should not be due to different task-relevant processing of emotional and neutral stimuli, but should reflect sustained emotional responding as indicated by considerable studies [47], [50], [64]. This argument was reinforced by our observation that the amplitudes of LPP, but not other components, were positively correlated with the arousal levels of the pictures (see Fig. 6).

We observed significant mood state by picture valence interactions on the LPP amplitudes of the 600–1000 ms interval, with the emotion effect for unpleasant pictures more pronounced during negative versus positive and neutral mood states. As indicated before, the present study used three different sets of pleasant, neutral and unpleasant pictures for the three mood blocks, which determined that the mood by picture valence interaction was unlikely due to practice/familiarity effects that are associated with repeated stimulus presentation [80]. Also, this interaction was unlikely to result from potential differences in picture valence or arousal across the three blocks, as the emotion attributes of each picture category were kept similar across the three blocks. As such, the emotion effect for unpleasant pictures should not have been different in the three blocks, if there was no additional impact from mood induction by presentation of music excerpts.

Using emotionally salient pictures, the present results displayed that negative mood intensified the brain's susceptibility to unpleasant events, as indexed by the larger size of LPP enhancement from neutral to unpleasant pictures during negative versus other moods. This finding was confirmed by our correlation analysis that showed larger enhancement of LPP emotional effect for unpleasant pictures with increasing self-reported negative mood (Fig. 7). Also, this result was in line with prior reports that affective disorders were associated with enhanced processing of negative stimuli, and that healthy subjects perceived more negative information from ambiguous faces during induced depression [20], [21]. The present findings, however, extended prior studies by showing that, apart from affective disorders which are linked with enhanced negative stimulus processing, the induction of negative mood state also enhances the brain's susceptibility to unpleasant events in healthy subjects.

According to this result, the role of affective disorder in negative stimulus processing is likely mediated by the predominant negative mood in the patients, such as various kinds of anxious or depressive disorders. This argument was reinforced by prior findings that state (but not trait) anxiety was associated with enhanced vigilance to threat stimulation [4], and that a therapy which remitted state but not trait anxiety reduced processing of negative stimuli [3]. However, we need to be cautious with this inference, as we did not include patient samples to directly examine whether mood states mediate the association between affective disorders and emotional stimulus processing. Furthermore, previous studies indicated a self-maintaining feature of affective disorders which directs patients' attention to the disorder-relevant stimuli. This bias in turn prolongs and intensifies the disorder [81], [82]. Similarly, the enhanced reaction to unpleasant events during negative mood is also likely to intensify negative mood itself; which might lead to a vicious circle harmful to psychophysical wellbeing. This possibility also needs to be directly examined in future studies.

On the other hand, positive mood, compared to neutral mood, was associated with decreased size of emotion effect for unpleasant pictures in the 800–1000 ms interval. This finding suggests that positive mood induction by happy music may be useful in dampening the intensity of emotion induced by negative stimuli. Also, this result was supported by prior findings of smaller attention bias for negative stimuli during positive vs. negative stimulus contexts [46]. It has been reported that positive affects increase broad-minded cognition which, in turn, promotes positive affects and wellbeing [83]. Therefore, the production of positive affect (by happy music, for example), is important to counteract negative emotional consequences and maintain wellbeing when facing unpleasant stimulation.

In addition, we did not observe a similar LPP enhancement effect for positive pictures during happy mood, but instead observed smaller LPPs for positive pictures during positive mood state (800–900 ms). Prior studies indicated that the brain's sensitivity to positive stimuli is susceptible to habituation, as it is biologically significant for organisms to keep approach motivation to new arousing event that meets different needs [84], [85]. This account was confirmed by an ERP study showing larger N1 amplitude reduction for positive versus negative stimuli during repeated stimulation [80]. Thus, our observation of reduced, instead of increased, LPPs to pleasant stimuli during positive mood, was probably a habituation effect due to antecedent perception of happy music. In contrast, it has been indicated that the human brain is equipped with sustained vigilance to aversive events, such that the brain kept attention vigilance for a negative stimulus presented for many times [80]. Moreover, it was indicated that negative music is powerful in inducing negative affect, and listening to negative music enhanced neural reactions to visual negative stimuli [86]. These factors might explain why negative mood increased the brain's reactivity to negative stimuli.

The present study did not observe a significant affective congruency effect in both 450–550 ms (P3) and 600–1000 ms (LPP) time intervals. As noted before, many affective priming studies have reported an emotional congruency effect during affective valence evaluation or prime-target affective congruency judgment tasks [41], [42], [51], [52]. Specifically, emotionally incongruent targets elicited delayed behavioral responses or a pronounced N400 effect 400–500 ms post target in comparison with emotionally congruent targets, when subjects were required to evaluate the valence of the target [41], [51], [53] or to judge the affective congruency of prime-target pairs [52]. However, the task in the present study required subjects to make an emotional/nonemotional classification for the picture stimuli, irrespective of the affective valence. By a series of experiments, Goerlich and colleagues [53] recently indicated that response competition is a necessary condition to elicit affective congruency effect. In a valence evaluation task, the affective valence of the target is a response-relevant dimension. Accordingly, the valence incongruence between prime and target produces response conflict that most likely accounts for the affective congruency effect in behavioral or electrophysiological levels. When subjects were engaged in object/person classification that was irrelevant to valence assessment, the affective priming effect was neither observed at behavioral nor electrophysiological levels [53]. Therefore, our use of a non-valence classification task most likely explained why we did not observe significant affective congruency effects in both components.

We need to acknowledge that the number of subjects (sixteen) recruited for our ERP study did not constitute a big sample size. Consequently, one may question the reliability of our key findings. However, the present study observed significant mood by picture valence interaction (i.e. negative mood enhanced LPPs for negative pictures compared to neutral mood) in four consecutive 100 ms time windows. Thus, this mood effect was reliably observed in different LPP phases. Also, we observed that the magnitude of this mood effect was larger with increasing negative mood arousal, reflected by a significant positive correlation between the two variables. Considering these converging evidences, the key findings of the present study should be statistically reliable, rather than being statistically vulnerable.

The present study used non-emotional, Mongolian language broadcasts to induce a neutral mood state. Our manipulation checks confirmed that these materials effectively induced a neutral mood state, as the mood ratings were similar during pre-experiment and neutral mood phases. However, language and music are distinct categories of stimuli. Thus, one may question the validity of using language stimuli as the baseline condition. A number of studies have reported that language and music processing shared behavioral and physiological characteristics. For example, it was indicated that musical and linguistic syntactical capacities were positively correlated [87]–[89]. Moreover, language and music production and perception were underlain by similar or overlapping neural substrates [90], [91]. For instance, Brown and colleagues [91] required subjects to improvise melodies or linguisitic sentences in response to unfamiliar, auditorily presented stimuli during brain imaging by positron emission tomography. The results showed that the two tasks revealed activations in nearly identical functional brain areas, including the primary and supplementary motor cortices, anterior insula, primary and secondary auditory cortices, temporal pole, basal ganglia, ventral thalamus, and posterior cerebellum, with the differences between melodic and sentential generation just seen in lateralization tendencies (the language task was left lateralized; [91]). These findings were later supported by the study by Schön and François [87] which observed similar ERP responses to linguistic and musical test-items. Also, these conclusions were supported by the findings of Miranda & Ullman [92], who observed similar effects during rule processing and memory processing in brain potentials across language and music. Consistent with these findings, Rowe and colleagues [32] used broadcast sentences to have successfully induced neutral mood state as compared to happy and sad moods induced by music. These abundant evidences suggest that it is valid to use language materials for inducing neutral mood state. However, considering language and music processing showed different patterns of cortical lateralization [91], carefulness should be exercised to interpret our current findings, and these findings need to be replicated using the same type of stimuli for inducing emotional and neutral mood states.

## Supporting Information

Appendix S1 **The number of CAPS pictures used for this study.**
(DOCX)
