# Supplementary material for: Negative Mood State Enhances the Susceptibility to Unpleasant Events: Neural Correlates from a Music-Primed Emotion Classification Task
Source: PLoS One. 2014 Feb 28;9(2):e89844. doi: 10.1371/journal.pone.0089844 (PMC3938531; doi:10.1371/journal.pone.0089844)
Supplement: Appendix S1 — The number of CAPS pictures used for this study. (DOCX) [file pone.0089844.s001.docx]

**Appendix S1:**

The number of CAPS pictures selected for the present study (The pictures and music excerpts used in this study are available by contact to the corresponding or first authors).

**Sad Block:**

Negative pictures:

533,443,159,185,191,194,196,206,218,222,232,246,281,283,484,522,

536,559,569,616

Neutral Pictures:

317,327,329,330,343,344,314,315,348, 311, 312, 356, 360, 367, 398, 401,406,424,425,434,477,479,631,705,711,717,719,726,732,767,793,803,808,828,830,834,836,837, 297,307

Positive Pictures:

718,11,12,15,16,28,40,57,83,101,102,461,472,473,478,640,109,675,684,762

**Neutral Block:**

Negative pictures:

188,193,199,205,240,244,248,273,276,282,284,471,554,555,573,580,590,611,627,629

Neutral pictures: 350,354,357,368,369,386, 346,347,407, 334,336,342, 410,419,459,465,698,702,713,722,724,733,735,739,746,772,777,778,

783,816,825,829,831,833,849, 292,293,295,301,319

Positive pictures:

6,14,18,20,52,39,53,94,129,448,457,661,488,701,10,781,800,813,814,822

**Happy Block:**

Negative pictures:

577,173,178,180,233,243,254,255,256,270,280,502,528,532,541,548,

583,584,605,624

Neutral pictures:

296,298,304,313,316,318,320,322,332,335,345,352,353,355,361,376,

403,455,464,481,495,534,649,682,693,709, 710,714,725, 741,742,747, 768, 785,810,826,832,839,843,851

Positive pictures:

641,775,13,43,45,65,88,113,117,118,121,430,432,437,453,650,679,

752,756,780
